# Interleukin-11 signaling underlies fibrosis, parenchymal dysfunction, and chronic inflammation of the airway

**DOI:** 10.1038/s12276-020-00531-5

**Published:** 2020-12-01

**Authors:** Benjamin Ng, Stuart A. Cook, Sebastian Schafer

**Affiliations:** 1 National Heart Research Institute Singapore, National Heart Centre Singapore, Singapore, Singapore; 2 Cardiovascular and Metabolic Disorders Program, Duke-National University of Singapore Medical School, Singapore, Singapore; 3 MRC-London Institute of Medical Sciences, Hammersmith Hospital Campus, London, United Kingdom; 4 National Heart and Lung Institute, Imperial College, London, United Kingdom

**Keywords:** Interleukins, Growth factor signalling

## Abstract

Interleukin (IL)-11 evolved as part of the innate immune response. In the human lung, IL-11 upregulation has been associated with viral infections and a range of fibroinflammatory diseases, including idiopathic pulmonary fibrosis. Transforming growth factor-beta (TGFβ) and other disease factors can initiate an autocrine loop of IL-11 signaling in pulmonary fibroblasts, which, in a largely ERK-dependent manner, triggers the translation of profibrotic proteins. Lung epithelial cells also express the IL-11 receptor and transition into a mesenchymal-like state in response to IL-11 exposure. In mice, therapeutic targeting of IL-11 with antibodies can arrest and reverse bleomycin-induced pulmonary fibrosis and inflammation. Intriguingly, fibroblast-specific blockade of IL-11 signaling has anti-inflammatory effects, which suggests that lung inflammation is sustained, in part, through IL-11 activity in the stroma. Proinflammatory fibroblasts and their interaction with the damaged epithelium may represent an important but overlooked driver of lung disease. Initially thought of as a protective cytokine, IL-11 is now increasingly recognized as an important determinant of lung fibrosis, inflammation, and epithelial dysfunction.

## Lung disease: suppressing immune protein could reduce fibrosis

An immune protein that contributes to fibrosis, inflammation, and cellular dysfunction in the airways should be a target of therapies for severe lung diseases. Previously thought of as protective, the cytokine interleukin-11 (IL-11) evolved as part of the body’s immune system, and is highly expressed in the parenchyma and stroma of the diseased lung. Sebastian Schäfer at National Heart Centre Singapore and co-workers review recent research evidence indicating that IL-11 signaling is centrally important in pathological processes including the dysfunction of epithelial cells, inflammation of connective tissue, and the activation of cells that induce fibrotic scarring. Antibody therapies targeting IL-11 can reduce pulmonary fibrosis and inflammation in mice. Pharmacological companies may soon begin clinical trials of anti-IL-11 therapies on patients with idiopathic pulmonary fibrosis, a severe lung disease sharing several risk factors with COVID-19.

## Introduction

The lung is a complex organ composed of epithelial, stromal, vascular, and immune cells that are critical for maintaining tissue function and repair. Healthy lungs have a remarkable capacity to regenerate after acute injury^1^. Chronic injury, on the other hand, can destroy tissue homeostasis, resulting in unresolved tissue damage and scarring, also known as fibrosis. Pulmonary fibrosis is a common pathology seen in numerous airway diseases, such as asthma, and after respiratory infections and is central to disease pathology in interstitial lung diseases. More recently, infections with severe acute respiratory syndrome coronavirus 2 (SARS-CoV-2) have also been associated with fibrosis. Notably, the major risk factors for COVID-19 overlap with those of idiopathic pulmonary fibrosis (IPF), the most common, severe, and lethal form of adult idiopathic interstitial pneumonia^2^.

IPF is a severe disease with a median survival of 3–5 years from the time of diagnosis^3^, and the incidence and mortality of IPF are on the rise^4^. Risk factors include increased age, smoking, viral infections, and genetic factors^5^. Currently, approved therapies (nintedanib and pirfenidone) reduce the decline in lung function and slow disease progression. However, despite treatment, the majority of IPF patients continue to deteriorate, and lung transplantation remains the only life-saving cure for this disease. The pathogenesis of pulmonary fibrosis in IPF is usually characterized by repetitive microinjury to the alveolar epithelium, followed by the recruitment and proliferation of fibroblasts that differentiate into activated myofibroblasts^6^. The role of inflammation in IPF is contentious.

Myofibroblasts are contractile and apoptosis-resistant cells that deposit excessive amounts of extracellular matrix (ECM) in the lung interstitium, leading to the replacement of normal lung tissue with collagenous scar tissue. Chronically activated epithelial cells and fibroblasts in the diseased tissue also express a variety of cytokines that trigger the recruitment and activation of immune cells. These cells, in turn, secrete various profibrotic proteins, inducing further fibroblast activation and ECM production in a vicious cycle^7^.

In the past several years, much progress has been made in uncovering the mechanisms underlying the complex process of lung tissue repair and scarring. However, our overall understanding of the various cytokines involved in inflammatory and fibrotic lung diseases remains incomplete. Here, we summarize the current and emerging roles of the cytokine interleukin (IL)-11 in the context of lung diseases and pulmonary fibrosis, with a focus on its central role in myofibroblast activation, and discuss the therapeutic potential of targeting IL-11 in IPF and other fibrotic lung diseases.

## IL-11 is a member of the IL-6 family of cytokines

IL-11 was discovered in 1990 and first described as a hematopoietic factor that stimulates plasmacytoma proliferation and megakaryocyte formation^8^. The dependence of IL-11 on the ubiquitously expressed receptor gp130^9^ designates IL-11 as a member of the IL-6 family of cytokines. IL-6 family cytokines have been shown to bind to gp130 alone^10–12^, although the accepted mode of signaling requires the interaction of cytokines with a specific, high-affinity receptor subunit (alpha chain) prior to binding to gp130^13^. The gp130 receptor subsequently triggers a number of different pathways, including canonical JAK/STAT signaling, as well as ERK and AKT^14,15^. Loss-of-function mutations in IL-6 family cytokines or receptors result in a diverse set of genetic disorders of varying severity^16–19^, which suggests nonredundant and distinct biological functions of members of this cytokine family. In the lung, IL-11 receptor subunit alpha (IL11RA) is highly expressed on cells such as fibroblasts, vascular smooth muscle cells, and epithelial cells^20–23^. These cell types are central for both acute and chronic diseases of the lung/respiratory tract. This receptor expression pattern differentiates IL11RA from the IL-6 receptor, which is most highly expressed on immune cells, including monocytes and tissue-resident macrophages^13^.

Several studies have identified individuals and families with homozygous or compound heterozygous loss-of-function mutations in IL11RA. Lifelong loss of IL-11 signaling is associated with craniosynostosis, joint laxity, scoliosis, and delayed tooth eruption^24–27^. A recent study suggested that these IL11RA-dependent developmental phenotypes might be less severe than originally reported, possibly due to ascertainment bias^28^. Interestingly, at the level of the general population, there appears to be no negative selection against predicted loss-of-function mutations in IL11RA, indicating that such mutations are not detrimental for replicative capacity and have not been selected against evolutionarily^29^. Mice with genetic deletion of IL11RA, in addition to exhibiting female infertility, mirror the skull and tooth phenotypes seen in humans^30^, which suggests that the function of IL11RA is conserved across species. Garbers and colleagues have confirmed this conclusion by introducing disease-associated human IL11RA mutations into mice, causing a craniosynostosis-like phenotype^31^.

## Stromal and parenchymal IL-11 secretion is a hallmark of infection and chronic airway disease

IL-11 appears to have evolved as part of the innate immune response in fish, where it is highly upregulated in various tissues in response to bacterial or viral infections^32^. IL-11 is secreted by tissue-resident cells that are not part of the immune system^33^ and is particularly elevated in moribund, nonsurviving fish after viral infection^34^. This mechanism appears to be conserved in humans, and IL-11 plays a role in viral airway disorders; human stromal cells stimulated with respiratory syncytial virus, rhinovirus, and parainfluenza virus type 3 secrete high levels of IL-11. In addition, nasal secretions and aspirations from children with upper respiratory tract infections contain elevated IL-11 protein levels, particularly in the context of bronchospasm^35^. Epithelial-like human cells (alveolar A549 and 9HTE airway cells) upregulate IL-11 in response to respiratory syncytial virus^36^. IL-11 was also shown to be elevated and plays a proinflammatory (and perhaps profibrotic) role in the lung following tuberculosis infection in genetically susceptible mice^37^. Moreover, IL-11 inhibition reduced the severity of tuberculosis-induced lung pathology and inflammation by inhibiting neutrophil influx and improved survival^37,38^. Recent data have also shown that IL-11 is temporally upregulated in the lung epithelium following acute *Escherichia coli* lung infection^39^. However, neither exogenous IL-11 treatment nor IL-11 inhibition in *E. coli-* or *Streptococcus pneumoniae-*infected mice had any impact on lung injury or pneumonia outcomes^39^, which suggests that IL-11 activity does not alter the initial immune responses and bacterial clearance in the context of acute respiratory infections.

In chronic inflammatory airway diseases, IL-11 is upregulated in epithelial cells in individuals with severe asthma, and its expression correlates with disease severity^40^. Th17 cytokines, which are increased in asthmatic airways, can induce IL-11 secretion by bronchial epithelial cells, bronchial fibroblasts, and eosinophils from asthmatic but not healthy individuals^41–43^. Additionally, an *IL11* promoter polymorphism is associated with the development of chronic obstructive pulmonary disease (COPD)^44^. Mouse models have also shown that the inducible expression of human IL-11 in utero in mice interfered with lung development and resulted in alveolar enlargement (emphysema)^45^. However, genetic deletion of IL11RA failed to ameliorate emphysema in a gp130 mutant mouse model of emphysema^46^.

These early studies showed that pulmonary stromal and epithelial cells can be a source of IL-11 as part of their response to pathogens. Considering that these cells also express high levels of IL11RA, IL-11 is likely to act in an autocrine and/or paracrine fashion in viral or inflammatory airway disease. Whereas fibroblasts transform into collagen-secreting myofibroblasts in response to IL-11 signaling^20–22^, epithelial cells appear to transdifferentiate into a mesenchymal-like phenotype^47^. Both mechanisms are dependent on noncanonical ERK signaling and result in alpha-smooth muscle actin (ACTA2) expression^47^.

The expression pattern of IL-11 in human pulmonary diseases is intriguing but does not reveal whether IL-11 upregulation is a protective or a disease-causing mechanism. Several studies have tried to elucidate the effects of IL-11 on the respiratory tract in vivo. Earlier studies showed that transgenic expression of human IL-11 in mice causes inflammation and airway remodeling^45,48^, but can also protect the lungs (and confer a strong mortality benefit in mice) from hyperoxia-induced DNA damage^49^. Administration of human IL-11 in rodents also reduced IgG immune complex-induced acute lung injury and pulmonary inflammation^50^ and was beneficial after LPS challenge^51^, but in contrast, genetic deletion of IL11RA protected against IL-13-driven airway inflammation and remodeling^52,53^. In hindsight, these experiments were confounded by the fact that species-foreign human IL-11 was expressed in the murine system. As we are now beginning to understand, human IL-11 triggers signaling pathways in a dissimilar fashion to endogenous murine IL-11 in mouse models. At high concentrations, human IL-11 can partially activate mouse fibroblasts^20^, but when human IL-11 is administered to mice, it can also act as an inhibitor of endogenous (pathogenic) murine IL-11 and thus reduce the activation of detrimental signaling pathways^54^. In addition, nonspecific inflammatory reactions may be triggered by expressing or injecting human IL-11 into rodents. This multitude of contrary effects of human IL-11 in the mouse makes it impossible to draw reliable conclusions about the role of IL-11 in the lung from earlier studies.

While IL-11 was initially implicated in pulmonary viral and inflammatory diseases, more recent studies have shown strong evidence that IL-11 is elevated in chronic lung diseases that have a fibrotic component. Stromal cells from scleroderma-associated interstitial lung disease patients highly express IL-11^55^, and IL-11 is also upregulated in the lungs of IPF patients and is positively correlated with fibrosis and negatively correlated with lung function^22,56^. Invasive IPF fibroblasts have been shown to secrete high levels of IL-11^57^. IPF patients with pulmonary hypertension (PH) have an even greater elevation in IL-11 and IL11RA expression in the lungs, which is associated with increased pulmonary artery remodeling compared to that of IPF patients without PH or non-IPF individuals^58^.

## IL-11-dependent ERK signaling drives myofibroblast differentiation and promotes cellular senescence in the lung

**Fig. 1:**
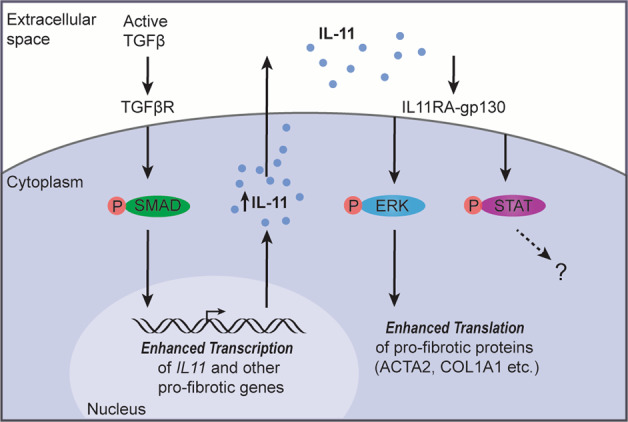
Autocrine IL-11 signaling underlies fibroblast activation. TGFβ stimulation of fibroblasts leads to canonical SMAD-dependent transcriptional activation, which upregulates *IL11* RNA and other profibrotic genes. IL-11 protein is secreted and binds to its cognate receptor (IL11RA) in an autocrine and/or paracrine fashion. The IL-11: IL11RA complex then signals via the ubiquitously expressed gp130 receptor to activate ERK to enhance the translation of profibrotic RNAs, which themselves can be elevated due to prior TGFβ-SMAD effects. IL-11, particularly at high concentrations, can also induce STAT3 phosphorylation. However, IL-11-related STAT activation has negligible effects on transcription in fibroblasts, and its relevance to fibrogenesis is unclear. ACTA2: Actin alpha 2, smooth muscle; COL1A1: collagen type I alpha 1 chain; ERK: extracellular signal-regulated kinase; gp130: glycoprotein 130, also known as interleukin 6 signal transducer; IL-11: interleukin 11; IL11RA: interleukin 11 receptor subunit alpha; STAT: Signal transducer and activator of transcription; SMAD: mothers against decapentaplegic homolog; TGFβ: Transforming growth factor-beta, TGFβR: Transforming growth factor-β receptor.

Myofibroblasts are the dominant source of collagen and ECM proteins and play a central role in the development of pulmonary fibrosis. The source of myofibroblasts in IPF has been a point of great debate and scrutiny for many years. Lineage tracing studies in mice have shown that these cells can arise from multiple cellular sources, including resident fibroblasts, pericytes, or circulating bone marrow-derived fibrocytes, following lung injury^59,60^. However, the precise mechanisms that drive myofibroblast differentiation and how these cells remodel the ECM and invade the parenchyma remain unclear^61^. It is possible that the process of myofibroblast differentiation is mediated by a combination of converging signaling pathways. In vitro studies of primary fibroblasts showed that TGFβ strongly induces *IL11* RNA and protein expression via canonical SMAD signaling^20^. IL-11 stimulation of lung fibroblasts upregulates profibrotic protein expression (ACTA2, COL1A1) and induces the phosphorylation of ERK and, to a lesser extent, STAT3^22^. IL-11 also induces the proliferation of normal and IPF-derived lung fibroblasts via an ERK-dependent mechanism^62^. However, in contrast to transforming growth factor-beta (TGFβ) stimulation, which has profound transcriptional effects, IL-11-stimulation of primary lung fibroblasts showed negligible differences in global RNA expression by RNA-seq analysis, indicating that IL-11-driven STAT3 phosphorylation may have little transcriptional consequence in this context. In support of a post-transcriptional role for IL-11 signaling in fibroblast activation, the profibrotic activities of IL-11 can be blocked by MEK/ERK inhibition^22^. Hence, a working model is that IL-11 drives lung fibroblast activation and fibrotic protein expression largely via a post-transcriptional mechanism (Fig. 1).

In addition to TGFβ, several other fibrogenic factors implicated in pulmonary fibrosis pathobiology, including platelet derived growth factor (PDGF), fibroblast growth factor 2 (FGF2), IL-13, oncostatin M (OSM), and endothelin-1, have been shown to induce IL-11 secretion from fibroblasts and to signal, in part, through the MEK/ERK pathway^22,63–66^. IL-11 inhibition attenuates the fibrogenic activities of these various profibrotic factors via specific reductions in ERK signaling^22^, suggesting that IL-11 may act as a point of signal convergence downstream of multiple stimuli.

A prominent pathological feature of IPF fibroblasts is their enhanced capacity to invade the ECM^67^. In a recent study that reported the transcriptomic response of invasive and noninvasive fibroblasts from IPF patients, *IL11* was found to be among the most upregulated genes in invasive cells, indicating a potential role of IL-11 in regulating fibroblast invasion^57^. In vitro gain- and loss-of-function studies from our laboratory confirmed that lung fibroblast chemotaxis and ECM invasion are both dependent on IL-11 signaling. Furthermore, IL-11 inhibition (using anti-IL-11 neutralizing antibodies) reduces the invasive capacity of IPF-derived fibroblasts and induces activated myofibroblasts to shift to a quiescent state^22^.

Cellular senescence is a state in which cells cease to divide and undergo phenotypic changes characterized by the secretion of senescence-associated secretory phenotype (SASP) factors. The accumulation of senescent cells and the persistence of the SASP leads to cellular dysfunction and chronic inflammation and appears to be important for the pathogenesis of IPF^6^. Fibroblasts from patients with IPF display characteristics of senescence and resistance to apoptosis^68^. In murine models of pulmonary fibrosis, pharmacological depletion of senescent cells improves lung function, suggesting a role of senescent cells in pulmonary fibrosis and IPF disease pathology^69^. Whether IL-11 plays a role in promoting senescence pathways in IPF has not been directly demonstrated. However, a study in 2003 showed that IL-11 inhibited Fas-induced apoptosis in IPF lung fibroblasts via the upregulation of BCL-2^62^. Compared to healthy fibroblasts, IPF-derived fibroblasts secrete increased amounts of SASP factors, including proinflammatory cytokines (e.g., IL-6 and IL-1β) and profibrotic mediators (e.g., FGF2 and TGFβ)^69^. Oxidative stress is another key component in the induction of cellular senescence and an important contributing factor in IPF pathogenesis^70,71^. As highlighted above, it is possible that a number of fibrogenic SASP factors may act via IL-11 to induce cellular senescence through paracrine interactions between senescent and nonsenescent cells. In support of this concept, a recent study by Chen et al. explored the contribution of TGFβ and IL-11 signaling in a mouse model of senescence-associated lung fibrosis due to *Bmi-1* deficiency and showed that TGFβ and IL-11 were both highly secreted by senescent lung fibroblasts and epithelial cells. The authors also demonstrated that IL-11-dependent ERK signaling promoted fibroblast senescence and triggered the epithelial-to-mesenchymal transformation of type II alveolar epithelial cells^47^. Given the emerging importance of epithelial dysfunction in IPF^72^, these results also point to a potentially critical but largely unexplored role of IL-11 in the lung parenchymal niche. A recent study also showed IL-11 upregulation in lung endothelial cells in bleomycin-induced aged mice, which points to a role for IL-11 in vascular dysfunction in the context of IPF^73^.

## Therapeutic targeting of IL-11 in fibrotic lung disease

Previous studies by our group suggested that IL-11 was highly fibrogenic in the cardiovascular system^20^ and the liver^21^ and given the abundance of data that showed that IL-11 was elevated in human fibrotic lung disease; we revisited IL-11 in the context of experimental lung fibrosis and IPF. In proof-of-concept studies, we showed that species-matched administration or fibroblast-specific overexpression of murine IL-11 in mice causes, rather than protects against, lung fibrosis^22^. Three weeks of daily treatment with recombinant murine IL-11 in mice led to significant accumulation of collagen I-expressing activated myofibroblasts in the lung interstitium, a corresponding upregulation in genes associated with pathological remodeling (such as *Col1a1*, *Timp1*, and *Mmp2*) and increased deposition of collagen in the lungs^22^. Likewise, in separate gain-of-function experiments, the overexpression of murine IL-11 in a fibroblast-restricted manner in *Il11*-transgenic mice crossed with *Col1a2*-CreERT mice caused widespread parenchymal disruption and collagen and ECM deposition in the lung. Notably, primary fibroblasts isolated from these fibroblast-specific *Il11*-overexpressing mice were found to be extremely invasive and exhibited the characteristics of IPF fibroblasts^22^.

Pharmacological inhibition of IL-11 signaling is beneficial and protective in an established murine model of IPF, the bleomycin-injured lung^22^. In genetic loss-of-function studies, we also showed that *Il11ra1*-knockout mice were protected from lung fibrosis^22^. Subsequent signaling studies in whole lung tissue from *Il11ra1*-knockout mice revealed that the protection against lung fibrosis was associated with a reduction in pathological ERK and, perhaps surprisingly, SMAD signaling. Early and late treatment with anti-IL-11 antibodies in bleomycin-challenged mice not only prevented but also reversed established pulmonary fibrosis and specifically reduced pulmonary ERK and SMAD signaling^22^. Notably, STAT3 activation was unaffected and therefore appears to be tangential to IL-11-mediated lung fibrosis. In parallel, the Snoek group generated several human lung organoid models of the genetic condition Hermansky-Pudlak syndrome (HPS), which is caused by mutations affecting lysosome-related organelles^23^. Expression profiling revealed that only mutations linked to a clinical subtype characterized by extensive fibrosis of the lung also drove high IL-11 expression in human epithelial cells. Deletion of *IL11* effectively reduced fibrosis in mutant organoids, suggesting that IL-11 is a therapeutic target in HPS patients suffering from lung fibrosis.

Therapeutic strategies aimed at inhibiting the inflammatory components of IPF, such as targeting IL-13, have to date failed in the clinic, and this raises the question of whether this approach addresses a key underlying pathology or a secondary phenomenon^74,75^. Interestingly, we found that by treating fibrosis with anti-IL-11 therapy, there was an additional anti-inflammatory effect, which we had not anticipated^22^. Considering that IL11RA is predominantly expressed in the stromal and parenchymal niches, it was surprising to us that IL-11 signaling directly or indirectly affected the inflammatory response in the injured lung. Not much is known about the relationship between tissue fibrosis and inflammation in the lung, and in IPF in particular. One potential connection between these pathologies is the activity of inflammatory fibroblasts, which have been shown to be central to the immune response in the joint and the gut^76–78^.

**Fig. 2:**
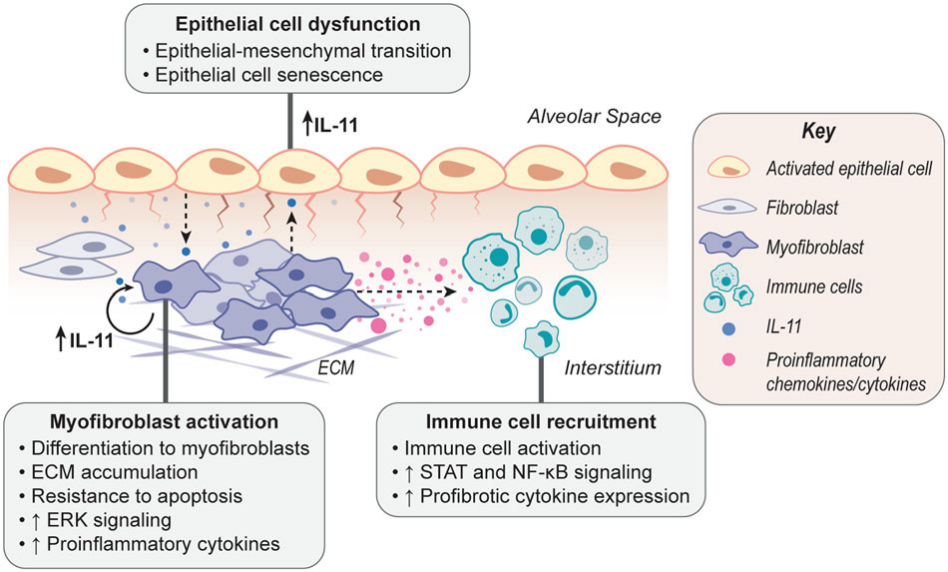
Schematic of IL-11 signaling in epithelial–stromal crosstalk and the development of pulmonary fibrosis. The damaged epithelium in diseased alveoli upregulates IL-11 and other factors, which in turn cause epithelial dysfunction by inducing epithelial–mesenchymal transition and senescence. Injury signals from the injured epithelium, including IL-11 itself, trigger IL-11 secretion from fibroblasts. IL-11 then acts in an autocrine/paracrine manner to drive/amplify profibrotic fibroblast effector functions, including proliferation, migration, invasion, and myofibroblast differentiation, via an ERK-dependent mechanism. IL-11-stimulated fibroblasts and myofibroblasts also acquire resistance to apoptosis and secrete extracellular matrix components, along with proinflammatory cytokines/chemokines (e.g., IL-6, CCL2, CXCL1), leading to the recruitment and activation of immune cells to the injury site. Inflammation perpetuates a vicious profibrotic loop that further activates fibroblasts. IL-11 expressed by fibroblasts may also contribute to paracrine activation of epithelial cells, causing chronic epithelial dysfunction and impaired regeneration.

To better understand the anti-inflammatory component of anti-IL-11 therapy, we generated a mouse with conditional deletion of the *Il11ra1* gene in fibroblasts. This allowed us to genetically block IL-11 signaling in vivo specifically in the stroma^79^. In vitro studies suggested that this inhibition would prevent fibroblast activation, migration, and ECM production. When we then induced the bleomycin model of pulmonary fibrosis, we observed a highly reduced fibrotic response (and ERK signaling) in the conditional knockout animals. While this effect on fibroblast activation was expected, we also observed reduced inflammation with fibroblast-specific IL11RA1 deletion. This effect was most notable in chronic, late-stage inflammation, whereas the immediate acute inflammatory response to injury was largely unaffected. Interestingly, inhibiting IL-11 signaling in fibroblasts led to specific reductions in profibrotic TGFβ-expressing monocyte and macrophage populations after bleomycin injury. This finding shows that the stroma has an upstream regulatory role in chronic inflammation that is a component of many chronic lung diseases and aging (Fig. 2)^80,81^. Hence, inflammation may be a secondary consequence of fibrosis and not a primary driver of disease, and targeting the activation of tissue-resident cells, such as fibroblasts or epithelial cells, may prove to be a more direct approach for treating fibroinflammatory lung diseases. This approach has the potential to reduce local inflammation without altering the systemic immune response, which may result in fewer side effects.

## Conclusion and future perspectives

Based on early studies of the effects of human IL-11 in mouse models of disease, IL-11 was considered (and is still thought) to be important for platelet function and to be cytoprotective, anti-inflammatory, and antifibrotic in organs, including the lung^82^. However, advances made in the past few years using species-matched IL-11 in disease models in vitro and in vivo have begun to suggest an alternative role for IL-11. IL-11 signaling is now known to be centrally important for lung pathology and promotes a broad range of maladaptive effects, including lung epithelial cell dysfunction, stromal-driven inflammation, and myofibroblast activation. Preclinical data have shown that IL-11 inhibition provides benefits in the treatment of certain respiratory infections and for the prevention and reversal of pulmonary fibrosis and inflammation. Studies using fibroblast-specific IL11RA1-knockout mice suggest that fibroblasts are a prominent cellular target of anti-IL-11 therapy. We believe that IL-11-dependent fibroinflammation may be broadly relevant across a range of chronic lung disorders.

Many questions relating to IL-11 biology in the lung remain. For example, the specific stimuli for IL-11 secretion in respiratory infections, asthma, and lung fibrosis are unclear. New methods for cell-type-specific lineage tracing coupled with single-cell sequencing technology may help uncover the major cellular sources of IL-11 in the diseased lung and further decipher the interactions between IL-11-expressing cells and other cell types during lung injury. Detailed mechanistic studies are also required to better understand how IL-11 affects lung epithelial and immune cells and how epithelial damage impacts stromal and immune activation. Signaling aspects also need to be fully deciphered. While ERK activity is central to IL-11-mediated effects in fibroblasts, the same may not be true in epithelial cells, and there are emerging links between IL-11 and NOX4 activity, reactive oxygen species, and additional signaling pathways.

Looking forward, anti-IL-11 therapies are currently being developed by pharmaceutical and biotechnology companies, and it is likely that at some point in the not-too-distant future, there will be clinical trials of anti-IL-11 therapy in patients with fibrotic and/or inflammatory lung diseases, such as IPF. Clinical trials for IPF are notoriously difficult, as there is great heterogeneity in patient lung function at presentation and in disease progression. It is perhaps reassuring that IL-11 levels in IPF correlate with disease severity; thus, targeting individuals with severe and/or progressive disease may define an opportune patient group characterized by high pulmonary IL-11 levels for clinical trials.

## References

[1] Basil MC (2020). The cellular and physiological asis for lung repair and regeneration: past, present, and future. Cell Stem Cell.

[2] George, P M. & Wells, A U. & Jenkins, R G. Pulmonary fibrosis and COVID-19: the potential role for antifibrotic therapy. *Lancet Respir. Med.* **8**, 807–815 (2020). 10.1016/S2213-2600(20)30225-3 PMC7228727 32422178

[3] Lederer DJ, Martinez FJ (2018). Idiopathic pulmonary fibrosis. N. Engl. J. Med..

[4] Hutchinson J, Fogarty A, Hubbard R, McKeever T (2015). Global incidence and mortality of idiopathic pulmonary fibrosis: a systematic review. Eur. Respir. J..

[5] Richeldi L, Collard HR, Jones MG (2017). Idiopathic pulmonary fibrosis. Lancet.

[6] Martinez FJ (2017). Idiopathic pulmonary fibrosis. Nat. Rev. Dis. Prim..

[7] Desai O, Winkler J, Minasyan M, Herzog EL (2018). The role of immune and inflammatory cells in idiopathic pulmonary fibrosis. Front. Med..

[8] Paul SR (1990). Molecular cloning of a cDNA encoding interleukin 11, a stromal cell-derived lymphopoietic and hematopoietic cytokine. Proc. Natl. Acad. Sci. USA.

[9] Yang YC, Yin T (1992). Interleukin-11 and its receptor. Biofactors.

[10] D’Alessandro F, Colamonici OR, Nordan RP (1993). Direct association of interleukin-6 with a 130-kDa component of the interleukin-6 receptor system. J. Biol. Chem..

[11] Rose-John S (1991). Structural and functional studies on the human interleukin-6 receptor. Binding, cross-linking, internalization, and degradation of interleukin-6 by fibroblasts transfected with human interleukin-6-receptor cDNA. J. Biol. Chem..

[12] Modrell B, Liu J, Miller H, Shoyab M (1994). LIF and OM directly interact with a soluble form of gp130, the IL-6 receptor signal transducing subunit. Growth Factors.

[13] Taga T, Kishimoto T (1997). Gp130 and the interleukin-6 family of cytokines. Annu. Rev. Immunol..

[14] Nguyen PM, Abdirahman SM, Putoczki TL (2019). Emerging roles for Interleukin-11 in disease. Growth Factors.

[15] Silver JS, Hunter CA (2010). gp130 at the nexus of inflammation, autoimmunity, and cancer. J. Leukoc. Biol..

[16] Dagoneau N (2004). Null leukemia inhibitory factor receptor (LIFR) mutations in Stüve-Wiedemann/Schwartz-Jampel type 2 syndrome. Am. J. Hum. Genet..

[17] Komori T, Tanaka M, Senba E, Miyajima A, Morikawa Y (2013). Lack of oncostatin M receptor β leads to adipose tissue inflammation and insulin resistance by switching macrophage phenotype. J. Biol. Chem..

[18] Masu Y (1993). Disruption of the CNTF gene results in motor neuron degeneration. Nature.

[19] Takahashi R (1994). A null mutation in the human CNTF gene is not causally related to neurological diseases. Nat. Genet..

[20] Schafer S (2017). IL-11 is a crucial determinant of cardiovascular fibrosis. Nature.

[21] Widjaja AA (2019). Inhibiting interleukin 11 signaling reduces hepatocyte death and liver fibrosis, inflammation, and steatosis in mouse models of non-alcoholic steatohepatitis. Gastroenterology.

[22] Ng, B. et al. Interleukin-11 is a therapeutic target in idiopathic pulmonary fibrosis. *Sci. Transl. Med.* **11**, eaaw1237 (2019). 10.1126/scitranslmed.aaw1237 31554736

[23] Strikoudis A (2019). Modeling of fibrotic lung disease using 3D organoids derived from human pluripotent stem cells. Cell Rep..

[24] Nieminen P (2011). Inactivation of IL11 signaling causes craniosynostosis, delayed tooth eruption, and supernumerary teeth. Am. J. Hum. Genet..

[25] Keupp K (2013). Mutations in the interleukin receptor IL11RA cause autosomal recessive Crouzon-like craniosynostosis. Mol. Genet. Genom. Med..

[26] Miller KA (2017). Diagnostic value of exome and whole genome sequencing in craniosynostosis. J. Med. Genet..

[27] Papachristoforou R, Petrou PP, Sawyer H, Williams M, Drousiotou A (2014). A novel large deletion encompassing the whole of the galactose-1-phosphate uridyltransferase (GALT) gene and extending into the adjacent interleukin 11 receptor alpha (IL11RA) gene causes classic galactosemia associated with additional phenotypic abnormalities. JIMD Rep..

[28] Brischoux-Boucher E (2018). IL11RA-related Crouzon-like autosomal recessive craniosynostosis in 10 new patients: resemblances and differences. Clin. Genet..

[29] Karczewski, K. J. et al. The mutational constraint spectrum quantified from variation in 141,456 humans. Nature **581**, 434–443 (2020). 10.1038/s41586-020-2308-7 PMC7334197 32461654

[30] Nandurkar HH (1997). Adult mice with targeted mutation of the interleukin-11 receptor (IL11Ra) display normal hematopoiesis. Blood.

[31] Agthe M (2018). Mutations in craniosynostosis patients cause defective interleukin-11 receptor maturation and drive craniosynostosis-like disease in mice. Cell Rep..

[32] Wang T, Holland JW, Bols N, Secombes CJ (2005). Cloning and expression of the first nonmammalian interleukin-11 gene in rainbow trout Oncorhynchus mykiss. FEBS J..

[33] Wangkahart E, Secombes CJ, Wang T (2018). Studies on the use of flagellin as an immunostimulant and vaccine adjuvant in fish aquaculture. Front. Immunol..

[34] Xu L, Podok P, Xie J, Lu L (2014). Comparative analysis of differential gene expression in kidney tissues of moribund and surviving crucian carp (*Carassius auratus**gibelio*) in response to cyprinid herpesvirus 2 infection. Arch. Virol..

[35] Einarsson O, Geba GP, Zhu Z, Landry M, Elias JA (1996). Interleukin-11: stimulation in vivo and in vitro by respiratory viruses and induction of airways hyperresponsiveness. J. Clin. Invest..

[36] Elias JA (1994). Epithelial interleukin-11. Regulation by cytokines, respiratory syncytial virus, and retinoic acid. J. Biol. Chem..

[37] Kapina MA (2011). Interleukin-11 drives early lung inflammation during *Mycobacterium tuberculosis* infection in genetically susceptible mice. PLoS One.

[38] Shepelkova G, Evstifeev V, Majorov K, Bocharova I, Apt A (2016). Therapeutic effect of recombinant mutated interleukin 11 in the mouse model of Tuberculosis. J. Infect. Dis..

[39] Traber KE (2019). Roles of interleukin-11 during acute bacterial pneumonia. PLoS One.

[40] Minshall E (2000). IL-11 expression is increased in severe asthma: association with epithelial cells and eosinophils. J. Allergy Clin. Immunol..

[41] Al-Muhsen S (2013). Th17 cytokines induce pro-fibrotic cytokines release from human eosinophils. Respir. Res..

[42] Molet S (2001). IL-17 is increased in asthmatic airways and induces human bronchial fibroblasts to produce cytokines. J. Allergy Clin. Immunol..

[43] Kawaguchi M (2009). IL-17F-induced IL-11 release in bronchial epithelial cells via MSK1-CREB pathway. Am. J. Physiol. Lung Cell. Mol. Physiol..

[44] Klein W (2004). A promotor polymorphism in the Interleukin 11 gene is associated with chronic obstructive pulmonary disease. Electrophoresis.

[45] Ray P (1997). Regulated overexpression of interleukin 11 in the lung. Use to dissociate development-dependent and -independent phenotypes. J. Clin. Invest..

[46] Ruwanpura SM (2011). Interleukin-6 promotes pulmonary emphysema associated with apoptosis in mice. Am. J. Respir. Cell Mol. Biol..

[47] Chen H (2020). TGF-β1/IL-11/MEK/ERK signaling mediates senescence-associated pulmonary fibrosis in a stress-induced premature senescence model of *Bmi-1* deficiency. Exp. Mol. Med..

[48] Tang W (1996). Targeted expression of IL-11 in the murine airway causes lymphocytic inflammation, bronchial remodeling, and airways obstruction. J. Clin. Invest..

[49] Waxman AB (1999). Targeted lung expression of interleukin-11 enhances murine tolerance of 100% oxygen and diminishes hyperoxia-induced DNA fragmentation. Chest.

[50] Lentsch AB (1999). Regulatory effects of interleukin-11 during acute lung inflammatory injury. J. Leukoc. Biol..

[51] Sheridan BC (1999). Interleukin-11 attenuates pulmonary inflammation and vasomotor dysfunction in endotoxin-induced lung injury. Am. J. Physiol..

[52] Chen Q (2005). IL-11 receptor alpha in the pathogenesis of IL-13-induced inflammation and remodeling. J. Immunol..

[53] Lee CG (2008). Endogenous IL-11 signaling is essential in Th2- and IL-13-induced inflammation and mucus production. Am. J. Respir. Cell Mol. Biol..

[54] Widjaja, A. A. et al. Redefining interleukin 11 as a regeneration-limiting hepatotoxin. http://www.bioRxiv.org/content/10.1101/830018v1, 10.1101/830018 (2019).

[55] Lindahl GE (2013). Microarray profiling reveals suppressed interferon stimulated gene program in fibroblasts from scleroderma-associated interstitial lung disease. Respir. Res..

[56] Bauer Y (2015). A novel genomic signature with translational significance for human idiopathic pulmonary fibrosis. Am. J. Respir. Cell Mol. Biol..

[57] Geng Y (2019). PD-L1 on invasive fibroblasts drives fibrosis in a humanized model of idiopathic pulmonary fibrosis. JCI Insight.

[58] Roger I, Estornut C, Ballester B, Milara J, Cortijo J (2019). Role of IL-11 in vascular function of pulmonary fibrosis patients. Eur. Respir. J..

[59] Hashimoto N, Jin H, Liu T, Chensue SW, Phan SH (2004). Bone marrow–derived progenitor cells in pulmonary fibrosis. J. Clin. Investig..

[60] Rock JR (2011). Multiple stromal populations contribute to pulmonary fibrosis without evidence for epithelial to mesenchymal transition. Proc. Natl. Acad. Sci. USA.

[61] Suganuma H, Sato A, Tamura R, Chida K (1995). Enhanced migration of fibroblasts derived from lungs with fibrotic lesions. Thorax.

[62] Moodley YP (2003). Inverse effects of interleukin-6 on apoptosis of fibroblasts from pulmonary fibrosis and normal lungs. Am. J. Respir. Cell Mol. Biol..

[63] Madala SK (2012). MEK-ERK pathway modulation ameliorates pulmonary fibrosis associated with epidermal growth factor receptor activation. Am. J. Respir. Cell Mol. Biol..

[64] Hardie WD (2010). Signaling pathways in the epithelial origins of pulmonary fibrosis. Cell Cycle.

[65] Elias JA (1994). IL-1 and transforming growth factor-beta regulation of fibroblast-derived IL-11. J. Immunol..

[66] Gallelli L (2005). Endothelin-1 induces proliferation of human lung fibroblasts and IL-11 secretion through an ET(A) receptor-dependent activation of MAP kinases. J. Cell. Biochem..

[67] Li Y (2011). Severe lung fibrosis requires an invasive fibroblast phenotype regulated by hyaluronan and CD44. J. Exp. Med..

[68] Álvarez D (2017). IPF lung fibroblasts have a senescent phenotype. Am. J. Physiol. Lung Cell. Mol. Physiol..

[69] Schafer MJ (2017). Cellular senescence mediates fibrotic pulmonary disease. Nat. Commun..

[70] Bargagli E (2009). Oxidative stress in the pathogenesis of diffuse lung diseases: a review. Respir. Med..

[71] Otoupalova E, Smith S, Cheng G, Thannickal VJ (2020). Oxidative stress in pulmonary fibrosis.. Compr. Physiol..

[72] Katzen, J. & Beers, M F. Contributions of alveolar epithelial cell quality control to pulmonary fibrosis. *J. Clin. Invest.* **130**, 5088–5099 (2020). 10.1172/JCI139519 PMC7524463 32870817

[73] Caporarello, N. et al. Vascular dysfunction in aged mice contributes to persistent lung fibrosis. *Aging Cell* **19**, e13196 (2020). 10.1111/acel.13196 PMC7431829 32691484

[74] Parker JM (2018). A phase 2 randomized controlled study of Tralokinumab in subjects with idiopathic pulmonary fibrosis. Am. J. Respir. Crit. Care Med..

[75] Idiopathic Pulmonary Fibrosis Clinical Research Network. (2012). Prednisone, azathioprine, and N-acetylcysteine for pulmonary fibrosis. N. Engl. J. Med..

[76] Dakin SG (2018). Pathogenic stromal cells as therapeutic targets in joint inflammation. Nat. Rev. Rheumatol..

[77] West, N. R. et al. Oncostatin M drives intestinal inflammation and predicts response to tumor necrosis factor-neutralizing therapy in patients with inflammatory bowel disease. *Nat. Med.* **23**, 579–589 (2017). 10.1038/nm.4307 PMC5420447 28368383

[78] Smillie CS (2019). Intra- and inter-cellular rewiring of the human colon during ulcerative colitis. Cell.

[79] Ng, B. et al. Fibroblast-specific IL11 signaling drives chronic inflammation in murine fibrotic lung disease. *FASEB J*. **34**, 11802–11815 (2020). 10.1096/fj.202001045RR 32656894

[80] Scott MKD (2019). Increased monocyte count as a cellular biomarker for poor outcomes in fibrotic diseases: a retrospective, multicentre cohort study. Lancet Respir. Med..

[81] Morse C (2019). Proliferating SPP1/MERTK-expressing macrophages in idiopathic pulmonary fibrosis. Eur. Respir. J..

[82] Cook SA, Schafer S (2020). Hiding in plain sight: interleukin-11 emerges as a master regulator of fibrosis, tissue integrity, and stromal inflammation. Annu. Rev. Med..

